# Computed tomography-guided cutting needle pleural biopsy: Accuracy and complications

**DOI:** 10.3892/etm.2014.2078

**Published:** 2014-11-18

**Authors:** YI-YUAN CAO, NA FAN, FEN XING, LI-YING XU, YAN-JUAN QU, MEI-YAN LIAO

**Affiliations:** Department of Radiology, Zhongnan Hospital of Wuhan University, Wuhan, Hubei 430071, P.R. China

**Keywords:** pleural biopsy, pleural disease, cutting needle, computed tomography-guided

## Abstract

In cases of pleural lesion, tissue samples can be obtained through thoracoscopy or closed needle biopsy for histopathological analysis. Cutting needle biopsy is a relatively recent addition to these techniques. The aim of this study was to evaluate the diagnostic accuracy and safety of computed tomography-guided cutting needle pleural biopsy (CT-CNPB), as well as the associated complications, in patients with pleural lesion. This study was a retrospective analysis of 92 percutaneous CT-CNPBs on 90 patients between March 2008 and May 2013. For group comparisons, χ^2^ tests were used to detect the risk factors for diagnostic accuracy (false-negative rate). Of the 92 CT-CNPBs, malignant lesions were diagnosed in 55 cases (mesothelioma in 12, metastatic pleural disease in 36, synoviosarcoma in one, indeterminate-origin disease in one and false-negative lesion in five) and benign pleural disease was diagnosed in 37 cases (inflammation in 15, tuberculosis in 10, granuloma in three, solitary fibrous tumor in two, hematoma in one, fungus in one and indeterminate-origin disease in five). The sensitivity of diagnostic malignant lesion was 90.9%, and the specificity and positive and negative predictive values were 100, 100 and 88.1%, respectively. The overall diagnostic accuracy was 94.6%. A specific diagnosis was achieved in 89.1% of malignant lesions and 86.4% of benign lesions. Univariate analysis of the risk factors affecting accuracy (false-negative rate) did not reveal any significant differences (all P>0.05). The complication rates were 6.5% for pneumothorax, 8.7% for hemorrhage and 1.1% for hemothorax. In conclusion, CT-CNPB is a safe and accurate diagnostic technique that can be recommended as the primary method of diagnosis in patients with pleural thickening or lesions observed by CT scan.

## Introduction

The diagnostic approach to pleural disease is a relatively neglected aspect of modern thoracic medicine, despite the fact that pleural disease affects ~300 subjects per 100,000 individuals per year worldwide (1,2). If a clinical suspicion of malignancy is high in patients with pleural effusion, cytological examination of pleural fluid samples is recommended; the diagnostic yield for malignancy with pleural cytology is 50–60%, which falls to 30% in effusions associated with malignant mesothelioma (MM) (3). Tumor type and the availability of reliable immunocytochemistry may influence the yield; for example, the cytological detection rate for adenocarcinoma is higher than that for squamous cell carcinoma, mesothelioma or lymphoma (4). When the distinction between primary and metastatic tumors is addressed, morphological criteria alone are not sufficient for a definite diagnosis of MM (5). In instances when cytology is non-diagnostic (3,4), closed percutaneous needle biopsy has traditionally been performed blindly using a reverse-beveled needle, such as an Abrams or Ramel needle; however, blind closed pleural biopsy has a relatively modest diagnostic yield of <60% for pleural malignancy (6).

Medical thoracoscopy enables the direct examination of the pleura and biopsies taken under direct vision and has a diagnostic yield superior to that of blind closed pleural biopsy and thoracocentesis. The diagnostic yield is 91–95% for malignant disease and can reach 100% for pleural tuberculosis. Furthermore, although medical thoracoscopy is more invasive and expensive, complications occur only infrequently (4,7). Despite this, the most efficient and cost-effective approach to pleural lesions remains unclear and controversial, particularly in cases requiring the acquisition of pleural tissue. Recent studies have proposed that image guidance may significantly increase the diagnostic yield while simultaneously decreasing the risk of complications. It has also been suggested that real-time computed tomography-guided cutting needle pleural biopsy (CT-CNPB), performed by a radiologist, is a promising technique for sampling the pleura, as it can improve diagnostic sensitivity to ~80% for pleural malignancy (8,9,10). Image-assisted biopsy is more likely to be diagnostic in the presence of pleural thickening >10 mm, pleural nodularity, pleural-based mass lesions of >20 cm and solid pleural tumors (7,11–16). In the present study, the diagnostic accuracy and safety of CT-CNPB was evaluated in patients requiring pleural tissue sampling.

## Materials and methods

### Study population

This study was a retrospective analysis of 92 percutaneous CT-CNPBs on 90 patients between March 2008 and May 2013. All procedures were performed by the same two radiologists who were experienced in performing CT-guided pleural biopsies. Percutaneous CT-CNPB was indicated in any patient with a plural lesion requiring biopsy. Patients excluded from the study included patients with lung-based tumors or no final diagnosis. Each patient provided their written informed consent prior to the procedure. This study was conducted in accordance with the Declaration of Helsinki and with approval from the Ethics Committee of Zhongnan Hospital of Wuhan University (Wuhan, China).

### Procedure

**Figure 1:**
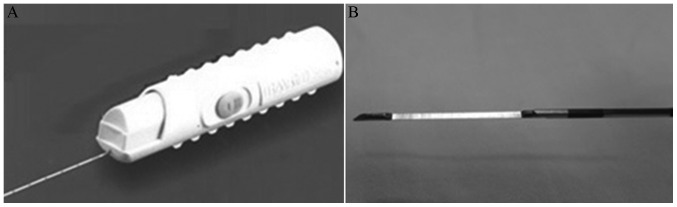
(A) Bard^®^ Max-Core^®^ 18 G biopsy needle; (B) top slide locked back and biopsy sample notch exposed.

**Figure 2:**
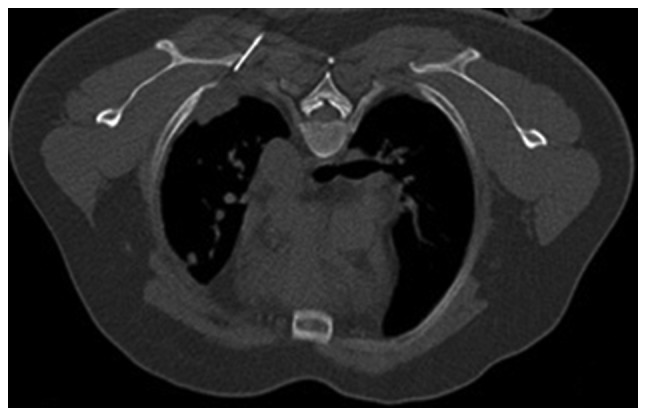
Male, aged 39 years, the first result of the computed tomography-guided cutting needle pleural biopsy was a false-negative lesion; the secondary biopsy led to a diagnosis of metastatic adenosquamous carcinoma.

**Figure 3:**
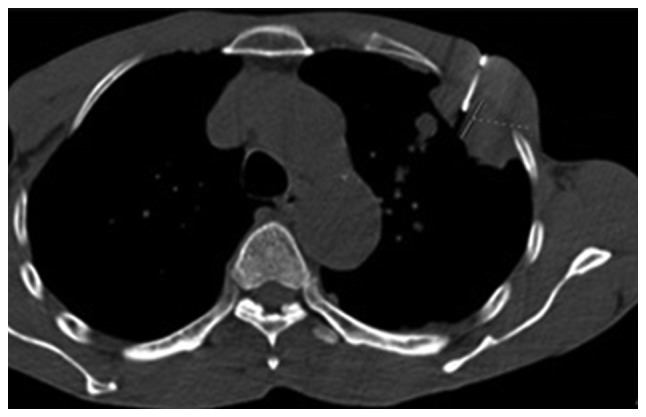
Male, aged 75 years, the result of the computed tomography-guided cutting needle pleural biopsy was malignant mesothelioma.

A CT scan of the chest (Somatom Sensation16; Siemens Healthcare, Forchheim, Germany) was initially performed to identify the lesion. The patient was then positioned in the supine, prone or lateral position to minimize puncture depth. An initial localization scan with a low-dose technique (Lung CARE series: 20–50 mA; 120 kV; scanning field, 30–60 mm; Siemens Healthcare) through the region of interest was performed at a slice thickness of 5 mm and viewed on both lung and soft-tissue windows. Localization was performed subsequent to the review of the CT images using laser positioning and skin markers (Biopsy single series: 50 mA; 120 kV; thickness, 10 mm; scanning field, 10 mm; Siemens Healthcare) to indicate the site of needle entry and the direction of approach for the biopsy. Subsequent to ensuring that the direction of needle approach was perpendicular to the chest wall, the thickness of the thoracic wall was measured from the skin marker to the pleural surface to determine the depth of anesthesia to be administered and the depth of needle insertion. Using an aseptic technique, local anesthetic (lignocaine 1%) was administered. An 18-gauge coaxial automated cutting needle (Bard^®^ Max-Core^®^ biopsy needle; C.R. Bard, Inc., Tempe, AZ, USA) (Fig. 1) was then introduced into the soft tissues without traversing the pleural surface (Figs. 2 and 3). The position of the pleural lesion in relation to the tip of the needle and the precise distance to the margin of the lesion (Figs. 2 and 3) were optimized using sequential CT scanning (Biopsy single series: 50 mA; 120 kV; thickness, 4.5 mm; scanning field, 13.5 mm; Siemens Healthcare). According to the precise information obtained from the repeat CT scan, the trajectory of the needle was adjusted, and the biopsy gun was directly advanced into the lesion and fired to obtain a core of tissue. The procedure was stopped immediately if the patient complained of discomfort, such as dyspnea, severe cough or hemoptysis. The surgeon then assessed the adequacy of the sample prior to deciding whether to proceed with additional passes. The obtained specimens were placed in formalin solution using a saline-filled syringe. Once the surgeon was satisfied with the samples obtained, immediate post-biopsy CT (Biopsy single series: 50 mA; 120 kV; thickness, 4.5 mm; scanning field, 13.5 mm) was performed over the region of the biopsy to check for pneumothorax or hemorrhage.

### Classification of diagnoses and complications

The biopsy specimens were evaluated by the same experienced pathologist. The cases were categorized primarily as benign and malignant, and those that were malignant were also categorized according to the cell properties. Immunohistochemical stains were used to differentiate tumors when required. The immunohistochemical markers used were: cytokeratin 7, thyroid transcription factor-1, vimentin, mesothelial cell and calretinin. A positive expression of calretinin, Vim and mesothelial cell would indicate that pleural mesothelioma occured, whereas, positive expression of cytokeratin 7 and thyroid transcription factor-1 would support that lung metastasis has occured. The pathologist also stained the specimens with Ziehl-Neelsen (Baso Diagnostics Inc., Zhuhai, China) to investigate for acid-resistant bacilli. A specific histological diagnosis was defined as a definite histological type; the results were considered non-specific when no particular diagnosis could be established from the specimen obtained and in false-negative cases of malignancy. The final diagnosis was confirmed at surgery. Histological findings obtained by biopsy were compatible with the patient’s clinical manifestations of disease.

The presence of pneumothorax was assessed by a low-dose CT technique. If the patient was clinically stable, he or she was kept under medical observation for 12 h. All patients had a chest radiograph performed 4 h after the procedure or sooner if they became symptomatic. Pneumothorax (17) and hemorrhage (18) were graded as mild, moderate or severe.

### Statistical analysis

Details regarding the nature of the lesion (lesion location and thickness), the procedure itself (false-negative rate, requirement for CT contrast enhancement to distinguish a lesion from the thorax wall and vessels and number of attempts) and complications were calculated. Categorical variables are presented as counts and percentages, and χ^2^ tests were conducted for group comparisons. Logistic regression models were performed to detect the risk factors for diagnostic accuracy (false-negative rate). Factors with a significance level of P<0.05 in the univariate analyses were included in the multivariate model. A two-sided P-value of <0.05 was considered statistically significant. Statistical Analysis System (SAS) version 9.2 statistical software (SAS Inc., Cary, NC, USA) was used for all analyses.

## Results

### Patient characteristics

In the present study, 90 patients, including 53 males and 37 females (mean age, 55.2±14.5 years) underwent 92 CT-CNPBs. There were 64 (71.1%) inpatients and 26 (28.9%) outpatients. Pleural thickening in the 92 cases varied between 6 and 99 mm, and 23 cases (25%) had pleural effusion. A total of 21 cases underwent CT contrast enhancement. The number of attempts ranged between two and four, and the duration of the procedure was 16±3 min.

### Biopsy yield

The distribution of diagnoses for cases included in the study is shown in Table I. Immunohistochemical stains were used in 19 cases. The sensitivity of diagnostic malignant lesion was 90.9% (50/55), the specificity was 100% (37/37), the positive predictive value was 100% (50/50) and the negative predictive value was 88.1% (37/42). The overall diagnostic accuracy was 94.6% (87/92). A specific histological diagnosis was achieved in 89.1% (49/55) of malignant lesions and 86.5% (32/37) of benign lesions. The non-specific results and confirmation methods are shown in Table II.

There were five false-negative cases in 55 malignant cases. Univariate analysis of the risk factors affecting accuracy (false-negative rate) did not reveal any significant differences (all P>0.05) (Table III).

### Complications

Pneumothorax occurred in six cases (6.5%) in the group with a pleural thickness of >30 mm; five (5.4%) of the cases were graded as mild and one case (1.1%) was graded as severe and required a chest drain. No further treatment was required for these cases. Mild lung hemorrhage around the entry site occurred in eight cases (8.7%) subsequent to the CT-CNPB. One patient (1.1%), who had a history of long-term oral aspirin administration, suffered from a hemothorax, which required tube thoracostomy. No cases required blood transfusion and no patients succumbed in the three days following the procedure. No patients required additional analgesics due to the pain.

## Discussion

Pleural diseases are a frequently occurring medical problem and the differential diagnosis is wide. Pleural aspiration is recommended as the first diagnostic procedure in patients with pleural effusion. This procedure is simple and safe and can often be performed at the bedside or in the clinic. The success rate of ultrasound-guided pleural aspirations can be ≤97% (16). For cases in which malignancy is suspected, fluid should be sent for cytological examination. The diagnosis of MM, metastasis or benign mesothelial proliferation in effusion samples is often challenging for medical professionals due to sampling problems (few malignant cells shedding and hemorrhagic or inflammatory effusion) and/or errors in interpretation. The reported sensitivity for the cytological diagnosis of MM ranges between 31.9 and 86.3% for malignancy without further specification and between 11.7 and 75.3% for a correct diagnosis of primary neoplasm (20). A significant proportion of biopsies may be technically inadequate and there is a significant false-negative rate of 35–50% (18). Furthermore, a percentage of false-positive cases still occur, with reactive mesothelial cells mimicking malignancy (5). In the present series, 25% of cases had pleural effusion.

The diagnostic yield of unaided (blind) closed pleural biopsy for pleural malignancy is a relatively modest <60%. Of note is the fact that the overall diagnostic yield for malignancy is only increased by 7–27% when compared with pleural fluid cytology (22). CNPB is a relative recent technique to be adopted. Image-guided cutting needle biopsy of mass lesions associated with pleural effusion is a well-validated modality, producing diagnostic yields higher than those for closed pleural biopsy. A contrast-enhanced thoracic CT scan of a patient with a pleural effusion may show focal areas of abnormal thickening (7). A study by Maskell *et al* (12) found that CT guidance significantly increased the diagnostic yield with regard to pleural thickening. In their study, CT-CNPB had a sensitivity of 87%, whereas unaided Abrams needle biopsy had a sensitivity of 44% (P=0.02) (12). Furthermore, Adams and Gleeson (24) previously found that CT-guided biopsies had a sensitivity of 93% for MM. The sensitivity of diagnostic malignant lesion calculated in the present study is among the highest of those previously published, and there were no false-positive cases in the group.

For pleural thickening of ≤5 mm, the sensitivity of CT-guided needle biopsy is 75%. The frequency of non-diagnostic biopsies ranges between 0 and 9% (11,25); therefore, if a CT-guided biopsy is performed in cases with minor pleural thickness, there may be a lower probability that a sufficient amount of tissue will be obtained. The rate of non-diagnostic pleural biopsies in the present series was 0%, and the rate of non-specific histological diagnosis was 12.0% (11/92). The non-specific cases may have been a result of inadequate biopsy samples and lesion complexity. In certain cases, biopsy would be insufficient for the final diagnosis; these cases would require further clinical and radiological investigations, as well as follow-up.

CT-guided biopsy may significantly increase the diagnostic yield while decreasing the risk of complications; the procedure can be performed in outpatient conditions and can be used for patients without pleural effusion. Furthermore, CT-CNPB can be performed in patients with pleural thickening in the absence of pleural fluid, which is not convenient when using an Abrams needle. The main complication associated with the procedure is pneumothorax. Although pneumothorax occurs in ≤15% of patients undergoing biopsies, very few require intervention. Other complications may include site pain (1–15%), vasovagal reaction with potential syncope (1–5%), hemothorax (<2%) and site hemorrhage with hematoma formation (<1%) (4). In the present series, the pneumothorax rate was 6.5%, and one patient required a chest drain. Bleeding occurred in 8.7% of the cases at the time of biopsy, although no subsequent blood transfusions were necessary. When an 17/18 or 20/21 G needle with a 2-cm throw is utilized to sample minimal pleural thickening in the absence of a pleural effusion, the visceral pleura and adjacent lung are likely to be traversed. A number of the observed pneumothoraces may have been a result of the introduction of air by the biopsy or drain rather than due to a direct communication with the airway.

Thoracoscopy has the advantage that it enables direct visualization of the pleura, although the visceral and parietal pleura must not be adherent for the technique to be performed. In a previous study, the CT-guided pleural Abrams needle biopsy group had a diagnostic sensitivity of 87.5%, whereas the medical thoracoscopy group had a sensitivity of 94.1% (P=0.252). Furthermore, CT-guided Abrams needle biopsy had a sensitivity of 95% in cases with pleural thickening ≥1 cm, which was similar to the sensitivity obtained with thoracoscopy (96%). Thoracoscopy achieved higher sensitivity in cases with <1 cm thickening (93 vs. 82%, P=0.42). The authors concluded that CT-guided Abrams needle biopsy should be used as the primary method of diagnosis in patients with pleural thickening or lesions observed by CT scan, but suggested that patients with the appearance of only pleural fluid on the CT scan may still benefit from primary medical thoracoscopy (14). Medical thoracoscopy has the advantage that it may additionally be used for therapeutic purposes, for example for the direct insufflation of talc in order to achieve pleurodesis and the breakdown of loculations. Surgical thoracoscopy, requiring complete deflation of a lung and superior access for therapeutic interventions, is significantly more invasive and expensive (4).

In conclusion, CT-CNPB is a safe and accurate diagnostic technique. It is suggested that the present method of CT-CNPB be used as a first diagnostic evaluation in those cases with pleural thickness or pleural lesion observed in the thoracic CT scan.

## Figures and Tables

**Table I:** Distribution of the diagnoses of the 92 cases included in the study.

A, Malignant, n=55 (59.8%)	

Diagnosis	N (%)
Mesothelioma	12 (13.0)
Metastatic pleural disease	36 (39.1)
Adenocarcinoma	24 (26.1)
Squamous carcinoma	5 (5.4)
Clear cell carcinoma	2 (2.2)
Adenosquamous carcinoma	1 (1.1)
Small cell carcinoma	1 (1.1)
Carcinocarcoma	1 (1.1)
Plasmocytoma	1 (1.1)
Osteosarcoma	1 (1.1)
Synovial sarcoma	1 (1.1)
Malignant cell	1 (1.1)
False-negative lesion	5 (5.4)

B, Benign, n=37 (40.2%)	

Diagnosis	N (%)

Inflammation	15 (16.3)
Tuberculosis	10 (10.9)
Granuloma	3 (3.3)
Solitary fibrous tumor	2 (2.2)
Hematoma	1 (1.1)
Fungus	1 (1.1)
Indeterminate-origin disease	5 (5.4)

**Table II:** Non-specific specimen results and the final diagnoses.

Case	Specimen results	Confirmation methods	Final diagnoses
1	Malignant cell	Secondary biopsy	Adenocarcinoma
2	Atypical mesothelial proliferation	Surgery	Adenosquamous carcinoma
3	Atypical mesothelial proliferation	Secondary biopsy	Adenocarcinoma
4	Fibrous tissue	Surgery	Adenosquamous carcinoma
5	Fibrous tissue	Clinical manifestation: Lesion enlarged	Metastatic pleural disease of lung adenocarcinoma
6	Fibrous tissue	Adenocarcinoma cell in hydrothorax	Metastatic pleural disease of ovarian adenocarcinoma
7/8	Glassy degeneration tissue and fibrous connective tissue	Clinical follow-up in two years	Benign lesion
9	Necrotic tissue	Absorbed after antituberculosis therapy	Tuberculosis
10	Fibrous connective tissue	Surgery	Inflammation
11	Fibrous tissue hyperplasia	Surgery	Solitary fibrous tumor

**Table III:** Univariate analysis of the risk factors affecting accuracy (false-negative rate).

	False-negative cases/total (%)	P-value
Gender		1.000
Male	3/31 (9.67)	
Female	2/24 (8.33)	
Age, years		0.878
≤50	1/14 (7.14)	
51–60	1/16 (6.25)	
61–70	2/14 (8.33)	
>70	1/11 (9.09)	
Lesion location		0.061
Right upper	0/10 (0.00)	
Right middle	0/2 (0.00)	
Right lower	1/18 (5.56)	
Left upper	0/11 (0.00)	
Left lower	4/14 (28.57)	
Lesion thickness, mm		0.144
≤10	2/6 (33.33)	
11–20	0/21 (0.00)	
21–30	1/6 (16.67)	
31–40	1/9 (11.11)	
>40	1/13 (7.69)	
Number of attempts		0.747
≤2	1/9 (11.11)	
3	3/40 (7.5)	
≥4	1/6 (16.67)	
Pleural effusion		0.649
Yes	1/19 (5.26)	
No	4/36 (11.11)	
Contrast enhancement		0.592
Yes	2/14 (14.29)	
No	3/41 (7.31)	
